# Reply: Faulty evidence for superconductivity in ac magnetic susceptibility of sulfur hydride under pressure

**DOI:** 10.1093/nsr/nwac087

**Published:** 2022-05-10

**Authors:** Xin Wang, Xiaoli Huang, Yiping Gao, Tian Cui

**Affiliations:** State Key Laboratory of Superhard Materials, College of Physics, Jilin University, Changchun 130012, China; State Key Laboratory of Superhard Materials, College of Physics, Jilin University, Changchun 130012, China; State Key Laboratory of Superhard Materials, College of Physics, Jilin University, Changchun 130012, China; State Key Laboratory of Superhard Materials, College of Physics, Jilin University, Changchun 130012, China; School of Physical Science and Technology, Ningbo University, China

**This reply is in response to 'Faulty evidence for superconductivity in ac magnetic susceptibility of sulfur hydride under pressure' by Hirsch (https://doi.org/10.1093/nsr/nwac086**.

In 2015, the discovery of high-temperature superconductivity in sulfur hydride (H_3_S) was reported with *T*_c_ of ≤203 K at 155 GPa, evidenced by electrical resistance and magnetization measurements [1,2]. This remarkable superconductivity was further verified by nuclear resonant scattering [3] and spectroscopic evidence [4]. Based on these results, in our previous work [5], we reported the changes in alternating current magnetic susceptibility due to superconductivity under variable pressures to map the superconducting phase diagram. Magnetic susceptibility measurements at high pressure are very challenging and greatly dependent on the sensitivity of the system and the size of the sample. At megabar pressures, the size of the sample is smaller than 100 × 100 × 10 μm^3^. Such a small sample has a weak magnetic signal change before and after superconductivity with several to tens of nV. By using a highly sensitive magnetic susceptibility system adapted for a megabar-pressure diamond anvil cell, we could achieve the aim to control the background lower than the sample signal. Therefore, it was possible to obtain the superconducting signal at megabar pressures on such a small sample.

Prof. Hirsch's letter discusses the temperature-changing rate and argues about its possible influence on superconducting transition during our magnetic susceptibility measurements. Prof. Hirsch plots Δ*T* versus temperature and finds the difference between subsequent measurements. In our experiments, we always set the rate of temperature change as a constant through the temperature controller. The constant and steady temperature change is realized by the continuous balance of the cooling gas and heating resistance. If cooling goes on, the cooling gas is occupying the main role, while the heating resistance plays the dominant role during the heating process. There is a common phenomenon that the rate of temperature change is not well controlled at low temperatures, especially close to the temperature limit of the cryostat. Therefore, the sudden changes in the rate occur at different temperatures unexpectedly. However, our present experiments indicate that such temperature breaks will not affect the detection of the superconducting signal at high pressures. Concurrently, the temperature breaks will not bring any new signal changes.

Prof. Hirsch specifically questions whether the superconducting *T*_c_ arises from the temperature break in the Δ*T*–*T* curve at 117 and 130 GPa. We think Prof. Hirsch ignores the chronological order of the superconductivity signal and temperature break. Actually, in our measurements, the sample data and background are collected from the heating process. The superconducting state exists at the low-temperature area while the normal state is located at the high-temperature range. The temperature break appears in the normal state after the superconducting transition. The magnetic signal appears while the temperature-changing rate is almost constant, as is shown in Fig. 1 and Supplementary Fig. S1. This is very important. At 117 GPa, with increasing temperature, the superconducting transition begins at 36.7 K and ends at 37.7 K, while the temperature break appears in the range of 37.9–38.4 K (see Fig. 1a–c). The situation is also same for 130 GPa (Supplementary Fig. S1). These data tell us that the transition from the superconducting into normal state occurs first and then the temperature break appears, and this means that such a signal is not caused by the temperature break. Therefore, our measured data demonstrate that there are no relationships between the superconducting transition signals and those temperature breaks. In addition, an important point should be noted that the superconducting transition is confirmed by magnetic signals whether there is a subtracted background or not (Fig. 1a and Supplementary Figs S1a–S6a).

**Figure 1.**
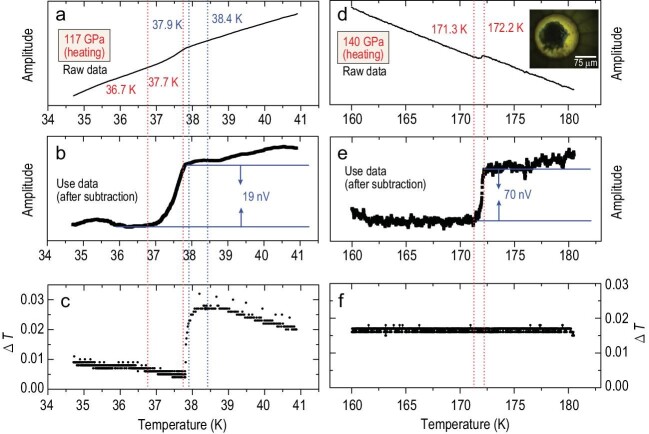
The magnetic susceptibility raw data, use data after subtracting the background and the change in the temperature of sulfur hydride at 117 GPa in (a)–(c) and at 140 GPa in (d)–(f). The red dotted lines indicate the superconducting transition region and the blue dotted lines show the beginning and ending points of the temperature break. The inset of (d) shows the sample chamber of the H_3_S sample directly synthesized from laser-heated mixtures of S and NH_3_BH_3_.

Besides those two pressure points, we also checked the data for 149 and 155 GPa (Supplementary Figs S2 and S3). It is clearly seen that the temperature change is normally fluctuating during the controlling of the temperature. At 149 GPa, at the end of this temperature range, the large temperature fluctuations come from the temperature-controlling feedback system of the cryostat. No evidence proves that the normally fluctuating temperature contributes to the superconducting transitions at the corresponding temperature range. Moreover, the temperature change or fluctuation could not be used to get information on the heat capacity of the system during the superconducting transition, which is a very complex situation for this cooling system and sample assembly. Very recently, we have detected high-temperature superconductivity in the H_3_S sample directly synthesized from laser-heated mixtures of S and H_2_ (H_2_ is generated from NH_3_BH_3_) [6,7]. In contrast to the direct compression of H_2_S at low temperatures, the use of NH_3_BH_3_ simplified the experimental procedure and significantly enlarged the sample of the final products. As illustrated in Fig. 1d–f, *T*_c_ of H_3_S sample is determined to be 172 K at 140 GPa with a much larger signal change. In this experimental run, there are no temperature breaks. All these data further strengthen confidence in high-temperature superconductivity of H_3_S.

To further verify our points, we also checked two typical superconductors MgB_2_ and Nb, of which superconductivity has been reported in the literatures [8,9], by using the present experimental method. Through the contrasting experiments with different rates of temperature change during heating, it is obvious that the superconducting transitions are triggered in these two samples. In the present experiment, we have loaded an MgB_2_ sample with a size of 100 × 100 × 35 μm^3^. First, in Fig. 2b, the temperature-changing rate is kept at 1 K/min and no temperature break is observed. The *T*_c_ of MgB_2_ is determined to be ∼39.3 K (Fig. 2a), consistently with previous measurements [8]. Second, we manually change the heating temperature rate during two stages: before and after the superconducting transition, and we get two temperature breaks during one heating run (Fig. 2d). But these breaks do not affect the detection of magnetic signals in both the superconducting and the normal states of the MgB_2_ sample, and the superconducting transition is still be detected at the same *T*_c_ (Fig. 2c). The similar situation can be found in the Nb sample with a size of 150 × 80 × 35 μm^3^ in Fig. 2e–h. Therefore, the present evidence shows that regardless of the existence of temperature breaks the same superconducting transition signal can be detected anyway. Importantly, the temperature breaks will not bring any new signals.

**Figure 2.**
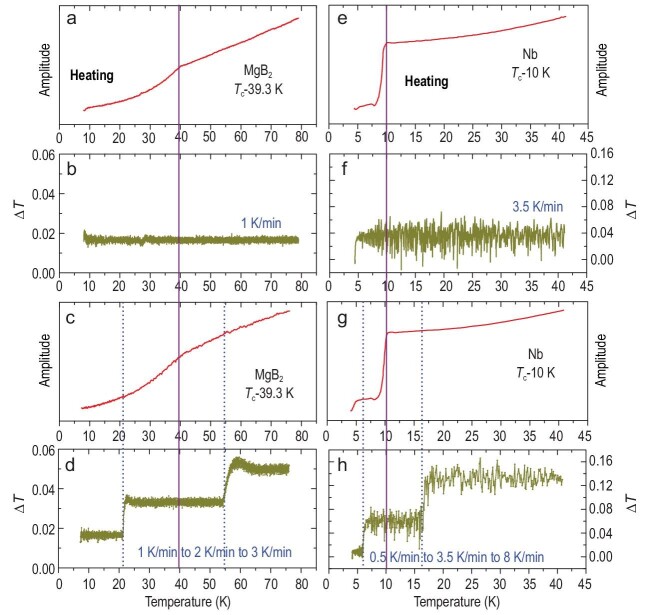
The magnetic susceptibility raw data and the change in temperature for MgB_2_ sample in (a–d) and Nb sample in (e–h), respectively. The purple solid lines indicate the superconducting *T*_c_ for MgB_2_ and Nb sample, and the blue dotted lines represent the emergence of the temperature breaks.

In addition, in our previous work [5], we also got repetition data and mapped the superconducting phase diagram for the high-temperature superconductor H_3_S (see Supplementary Figs S4−S6). The target H_3_S sample was prepared using a low-temperature compression path and the maximum *T*_c_ was observed at 183 K and 149 GPa [5]. For the low *T*_c_ phase at <140 GPa, the calculated *T*_c_ of the various stoichiometries of the H–S phase may be responsible for the results [5,10,11]. In contrast, the high *T*_c_ phase is mainly composed of the cubic H_3_S phase and pressure-dependent *T*_c_ is also consistent with the earlier theoretical calculation [12]. Besides, it is worth noting that Eremets *et al.* also report new evidence of the Meissner effect in high-temperature superconducting H_3_S using a superconducting quantum interference device (SQUID) [13] and they determine *T*_c_ ∼ 196 K in the *Im*-3*m*-H_3_S phase at 155 GPa.

In summary, all the present experimental results are enough to prove the high-temperature superconductivity in H_3_S under high pressure [1–5,13]. As is known to all, the measured accuracy of the physical parameters at high pressure is greatly affected by the sample dimensions, in contrast to the measurements at ambient pressure. The real useful and effective signal can be better obtained at high pressure with the development of technology, showing the new high-pressure physics accordingly.

## Supplementary Material

nwac087_Supplemental_File
