# Supplementary material for: Reply: Faulty evidence for superconductivity in ac magnetic susceptibility of sulfur hydride under pressure
Source: Natl Sci Rev. 2022 May 10;9(6):nwac087. doi: 10.1093/nsr/nwac087 (PMC9249580; doi:10.1093/nsr/nwac087)
Supplement: nwac087_Supplemental_File [file nwac087_supplemental_file.docx]

**Supplemental Material**

**for**

**Reply: Faulty evidence for superconductivity in ac magnetic susceptibility of sulfur hydride under pressure**

Xin Wang, Xiaoli Huang, Yiping Gao and Tian Cui*

*State Key Laboratory of Superhard Materials, College of Physics, Jilin University, Changchun 130012, People’s Republic of China.*

*To whom correspondence should be addressed: cuitian@jlu.edu.cn

**All the data published in our previous paper (Ref. 5, Nat. Sci. Rev. 2019, 6, 713-718), as follows:**





**Figure S1**. The magnetic susceptibility raw data (a), use data after subtracting the background (b) and the change in temperature heating rate (c) of sulfur hydride at 130 GPa. The red solid lines indicate the superconducting transition region, and the blue dash lines show the beginning and ending points of the temperature break.


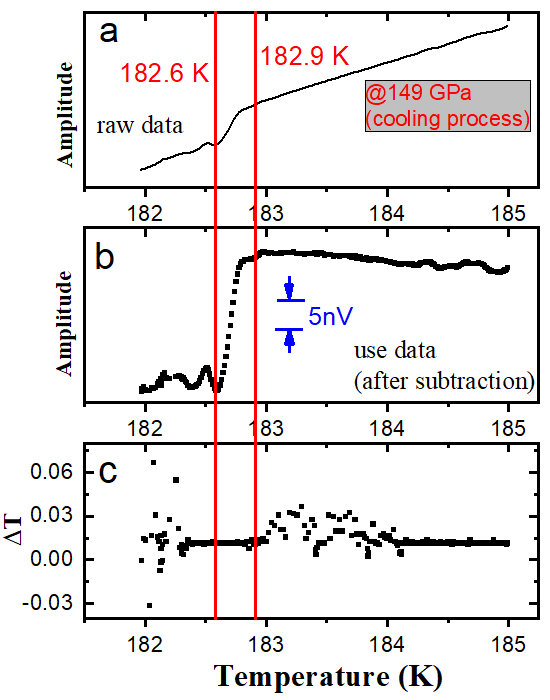


**Figure S2**. The magnetic susceptibility raw data (a), use data after subtracting the background (b) and the change in temperature cooling rate (c) of sulfur hydride at 149 GPa. The red solid lines indicate the superconducting transition region.


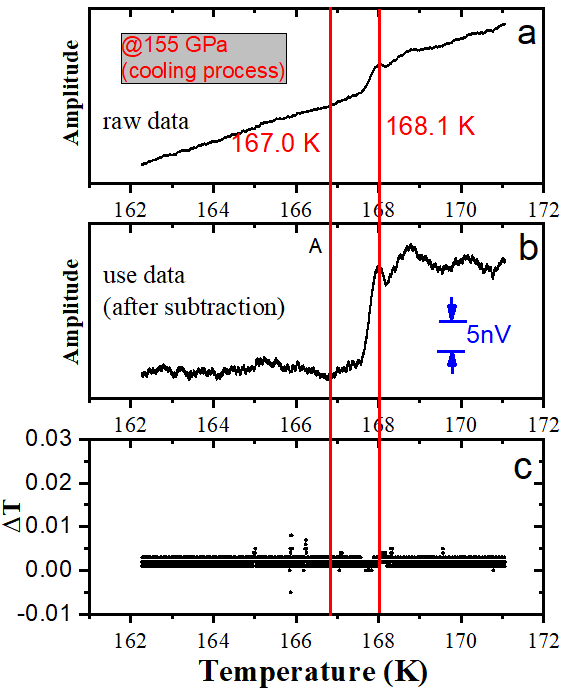


**Figure S3**. The magnetic susceptibility raw data (a), use data after subtracting the background (b) and the change in temperature cooling rate (c) of sulfur hydride at 155 GPa. The red solid lines indicate the superconducting transition region.


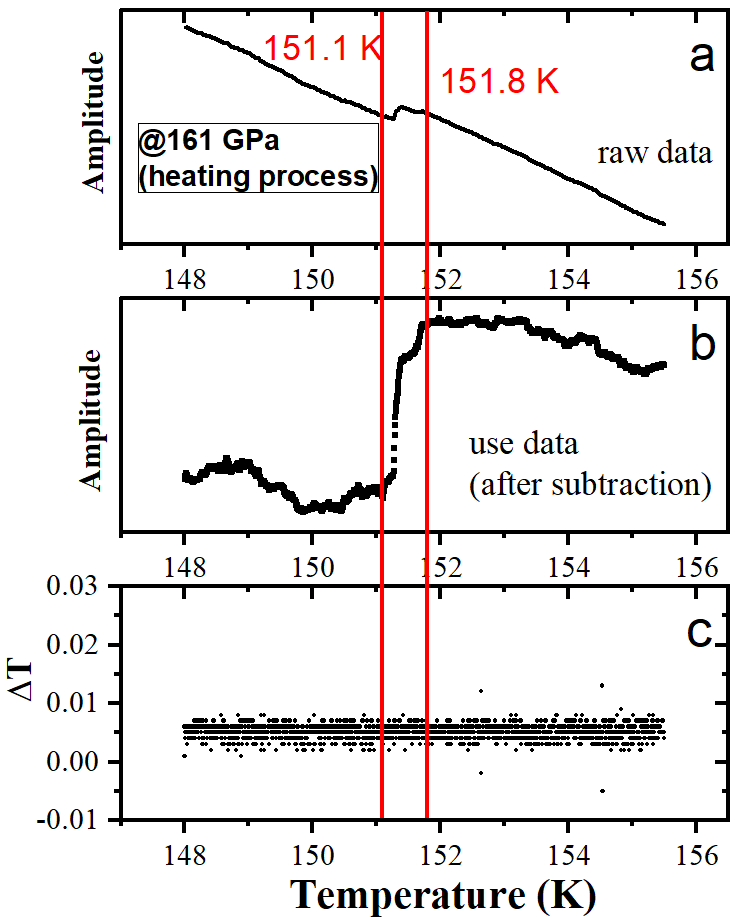


**Figure S4**. The magnetic susceptibility raw data (a), use data after subtracting the background (b) and the change in temperature heating rate (c) of sulfur hydride at 161 GPa. The red solid lines indicate the superconducting transition region.





**Figure S5**. The magnetic susceptibility raw data (a), use data after subtracting the background (b) and the change in temperature cooling rate (c) of sulfur hydride at 143 GPa. The red dash lines indicate the superconducting transition region.





**Figure S6**. The magnetic susceptibility raw data (a), use data after subtracting the background (b) and the change in temperature heating rate (c) of sulfur hydride at 171 GPa. The red dash lines indicate the superconducting transition region.

**Experimental Methods for getting new data (Fig. 1d-1f)**

In the present new repeated experiment, we have chosen diamond anvil cells (DACs) with 150 μm culets to achieve one megabar pressure. DACs were made of a high-purity Be-Cu alloy. Rhenium gasket was preintended to a thickness of 20-30 μm, and a hole with a diameter of about 80 μm was drilled using a laser as the sample chamber. The samples were prepared by sandwiching thin plates of 10-μm-thick sulfur between two 10-μm-thick layers of NH_3_BH_3_. NH_3_BH_3_ acted both as a source of H_2_ and a thermal isolator from the diamonds during laser heating. Subsequently, loaded DACs were increased to a desired pressure (approximately above 140 GPa), and then laser heated to ~1700 K.

For magnetic measurements, the 180 turns pickup and compensating coil are wound with 38 μm insulated copper wire, with the inner diameter being 3 mm. Two 180 turns exciting coils are wound on the pickup and compensating coil for generating alternating current magnetic field. The exciting coils are driven by an alternating current. The sample is in the pickup coil. Except of the signal of sample, the signals of pickup and compensation coils change almost synchronously during cooling and warming. This allows the signal from the sample to be separated from that coming from the background. The signal changes abruptly in the vicinity of the superconducting transition, allowing us to see these changes in the amplitude of the signal.
